# Supplementary material for: Genome-wide CRISPR Screens in T Helper Cells Reveal Pervasive Crosstalk between Activation and Differentiation
Source: Cell. 2019 Feb 7;176(4):882–896.e18. doi: 10.1016/j.cell.2018.11.044 (PMC6370901; doi:10.1016/j.cell.2018.11.044)
Supplement: Data S2. Processed Data from All the Steps of the Analysis, Related to Figure 1 [file mmc2.zip › supplemental data/motif analysis/Stat6_SRX021632_homer/homerResults/motif4.similar.html]

motif4

## Information for motif4

G
C
A
T
G
A
C
T
T
G
A
C
G
A
C
T
G
A
T
C
C
T
A
G
A
C
T
G
C
T
G
A
C
T
G
A
T
G
C
A
  
Reverse Opposite:  

A
C
G
T
G
A
C
T
G
A
C
T
T
G
A
C
G
A
T
C
C
T
A
G
C
T
G
A
A
C
T
G
C
T
G
A
C
G
T
A
  

|  |  |
| --- | --- |
| p-value: | 1e-936 |
| log p-value: | -2.157e+03 |
| Information Content per bp: | 1.710 |
| Number of Target Sequences with motif | 4357.0 |
| Percentage of Target Sequences with motif | 17.23% |
| Number of Background Sequences with motif | 1349.7 |
| Percentage of Background Sequences with motif | 5.59% |
| Average Position of motif in Targets | 101.8 +/- 42.7bp |
| Average Position of motif in Background | 99.7 +/- 60.3bp |
| Strand Bias (log2 ratio + to - strand density) | -0.0 |
| Multiplicity (# of sites on avg that occur together) | 1.10 |
| Motif File: | file (matrix) reverse opposite |

### Similar de novo motifs found

|  |  |  |  |  |  |  |  |
| --- | --- | --- | --- | --- | --- | --- | --- |
| Rank | Match Score | Redundant Motif | P-value | log P-value | % of Targets | % of Background | Motif file |
| 1 | 0.715 | C G T A A C G T G A C T A G C T G A T C G A T C G C A T C T A G T A C G A G C T C T G A T G C A | 1e-172 | -397.546161 | 2.46% | 0.63% | motif file (matrix) |
| 2 | 0.827 | A G C T G T A C G A C T C T G A T C G A C A T G G C T A T C G A | 1e-139 | -322.224115 | 5.79% | 2.81% | motif file (matrix) |
